# The Impact of Cardiopulmonary Bypass on the Structure and Mechanics of Red Blood Cells: Pilot Study

**DOI:** 10.3390/jcm15041435

**Published:** 2026-02-12

**Authors:** Viktoria Sergunova, Boris Akselrod, Snezhanna Kandrashina, Denis Guskov, Mikhail Shvedov, Olga Dymova, Alexander Grechko, Maxim Dokukin, Ilya Eremin, Vladimir Inozemtsev, Artem Kuzovlev, Ekaterina Sherstyukova

**Affiliations:** 1 Federal Research and Clinical Center of Intensive Care Medicine and Rehabilitology, V.A. Negovsky Research Institute of General Reanimatology, 107031 Moscow, Russia; 2 Petrovsky National Research Centre of Surgery, The Federal Agency for Scientific Organizations, 119991 Moscow, Russia

**Keywords:** cardiopulmonary bypass, hypothermic circulatory arrest, red blood cells, morphology, membrane, atomic force microscopy (AFM), Young’s modulus

## Abstract

**Background/Objectives**: Cardiopulmonary bypass (CPB) facilitates complex cardiac surgery but can damage erythrocyte membranes, impairing microcirculation and oxygen transport. Standard rheological tests assess overall blood properties but fail to define specific cellular mechanisms. In this study, atomic force microscopy (AFM) was employed to characterize morphological, nanostructural, and mechanical changes in erythrocytes following CPB and CPB combined with hypothermic circulatory arrest (HCA). **Methods**: The study included 14 patients who underwent cardiac surgery with CPB. Patients were divided into two groups. Group 1 underwent heart valve surgery with normothermic CPB (*n* = 7), and Group 2 underwent aortic arch surgery with CPB combined with HCA and moderate hypothermia (28 °C) (*n* = 7). Arterial blood samples were collected before the induction of anesthesia and immediately after CPB. The morphology and surface roughness (Rtm) of the erythrocyte membrane were evaluated on air-dried blood smears. Young’s modulus (E) was estimated from force-distance curves on living cells; measurements were performed at 24 °C in PBS. **Results**: Following CPB, both groups exhibited a decrease in the proportion of discocytes and an increase in echinocytes. In the CPB+HCA group, discocytes were absent after surgery. The mean Rtm increased 1.4-fold in Group 1 and 1.6-fold in Group 2, indicating greater nanostructural membrane damage in the latter. In Group 1, Young’s modulus increased by an average of 1.6 times, indicating increased cell stiffness. In Group 2, the increase was smaller (mean: 1.1 times) and was not statistically significant in some patients. **Conclusions**: Normothermic CPB primarily affects the nanomechanical properties of erythrocytes, whereas CPB+HCA induces more severe morphological and membrane surface damage while relatively preserving cytoskeletal elasticity. AFM-derived parameters of membrane roughness and cell elasticity may serve as sensitive indicators of erythrocyte biophysical integrity.

## 1. Introduction

Modern cardiopulmonary bypass (CPB) techniques enable complex reconstructive procedures on the heart and major vessels but are associated with several adverse effects, as CPB significantly impacts blood cellular components. One of the major complications is red blood cell (RBC) damage caused by shear stress and the activation of inflammatory and oxidative responses [1,2,3,4].

During aortic arch surgery, hypothermic circulatory arrest (HCA) with antegrade cerebral perfusion is combined with deep or moderate hypothermia. These strategies protect organs from ischemia [5,6,7]. However, despite their protective benefits, these procedures can negatively affect blood cells, leading to structural alterations, osmotic imbalances, and reperfusion injury [8].

While some damage leads to complete RBC destruction, other forms are limited to the membrane level, where the cell remains intact, but its membrane undergoes structural and functional changes [9,10]. These changes include not only reduced deformability and increased aggregability but also nanostructural alterations, which lead to increased surface roughness. The condition of the red blood cell membrane influences its deformability, its ability to pass through microvessels, and its circulation time [6,11]. Disruption of membrane integrity and nanostructural changes can impair microcirculation and reduce the blood’s oxygen transport function [12,13], which is critical for patients with cardiovascular disease.

If these alterations are mild, they may be partially reversible. However, with significant or prolonged exposure, the damage becomes permanent [14]. Erythrocyte membrane changes are difficult to detect using standard laboratory methods, yet it remains clinically essential. Reduced deformability and nanostructural damage impair the ability of cells to pass through microvessels and limit microcirculation and oxygen transport [15]. Stiffer and structurally altered RBCs have a lower capacity for oxygen saturation in the lungs, worsening tissue hypoxia [16,17]. Moreover, these changes promote the faster removal of damaged cells by the spleen and can result in ischemic organ damage due to impaired tissue perfusion [18,19].

Atomic force microscopy (AFM) enables comprehensive nanoscale characterization of red blood cells, including membrane topography and nanomechanical properties [20]. These parameters not only help identify the extent of cell damage but also suggest potential mechanisms of its development based on operating conditions and therapy.

Most research on the effects of cardiopulmonary bypass and circulatory arrest has focused on blood rheological properties. However, these approaches reflect the overall effect, including plasma and blood cells, without allowing differentiation of each component’s role. The aim of this study was to examine morphological, nanostructural, and mechanical changes in red blood cells following CPB and CPB combined with HCA procedures.

## 2. Materials and Methods

**Figure 1:**
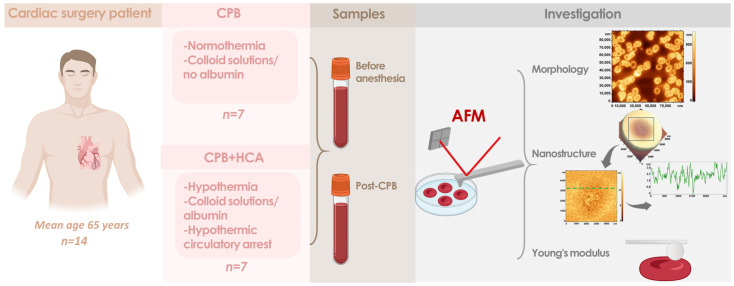
A schematic overview of the experimental procedure. Patients were divided into two groups: (1) those undergoing cardiopulmonary bypass (CPB) and (2) those undergoing CPB with hypothermic circulatory arrest (HCA). Blood samples were collected before the induction of anesthesia and after CPB. Red blood cells were examined using atomic force microscopy to assess their morphology, nanostructure, and Young’s modulus.

Figure 1 provides a schematic overview of the experimental procedure.

### 2.1. Patients and Study Groups

The study involved 14 patients who underwent cardiac surgery with cardiopulmonary bypass. Patients were divided into two groups:

Group 1 (CPB): Patients who underwent heart valve surgery with CPB and normothermic perfusion (35 °C) (*n* = 7).

Group 2 (CPB+HCA): Patients who underwent aortic arch surgery using CPB and antegrade cerebral perfusion combined with hypothermic circulatory arrest at 28 °C (*n* = 7).

Clinical characteristics of the patients: Group 1 included patients with aortic valve pathology requiring surgical correction. Group 2 included patients with pathology of the ascending aorta and aortic arch, specifically an aneurysm, which was an absolute indication for surgical treatment. Exclusion criteria were severe heart failure, acute inflammatory diseases, disorders of the blood and hematopoietic system such as anemia and leukemia, and the use of antiplatelet agents or anticoagulants before surgery.

In both groups, extracorporeal circulation systems Affinity NT (Medtronic Inc., Minneapolis, MN, USA) were used. None of the patients required ultrafiltration.

In Group 1, the mean patient age was 62.3 ± 8.8 years (2 men, 5 women). In Group 2, the mean age was 67.3 ± 5.6 years (6 men, 1 woman).

For Group 1, the CPB duration was 82 ± 18 min, with the administration of 1200 mL of a standard balanced solution, consisting of 700 mL balanced crystalloid and 500 mL colloid solutions.

For Group 2, the CPB duration was 126 ± 13 min, and the HCA duration was 28 ± 2 min. All patients in this group received 700 mL of balanced crystalloid solutions, 500 mL of colloid solutions, and 100 mL of 20% albumin.

The initial priming volume of the cardiopulmonary bypass circuit included a succinylated gelatin solution “Gelofusine” (B. Braun Medical AG, Sempach, Switzerland). No colloid solutions were administered at other stages of the study.

Written informed consent was obtained from all participants. The study was approved by the Local Ethics Committee of the Petrovsky National Research Centre of Surgery (Protocol No. 3, 21 March 2025). The study was conducted in accordance with the ethical standards of the Declaration of Helsinki of the World Medical Association.

### 2.2. Collection and Preparation of AFM Samples

Arterial blood samples (2.7 mL) were collected in K3-EDTA tubes (Sarstedt AG and Co. KG, Nümbrecht, Germany) from patients before the induction of anesthesia (preoperative sample) and immediately after CPB (Figure 1).

### 2.3. Preparation of Samples for Morphology and Nanostructure Analysis

From each sample, 9 µL of whole blood was aliquoted, applied to a microscope slide, and air-dried. A blood smear was then prepared using a V-Sampler machine (Vison, Perchtoldsdorf, Austria), after which the samples were imaged using AFM.

### 2.4. Preparation of Samples for AFM Force Spectroscopy

Blood samples were centrifuged to separate red blood cells from plasma and white blood cells. On the next step, an RBC suspension was prepared by adding 5 µL of RBCs to 1 mL of PBS. From this suspension, 100 µL was taken and diluted in 900 µL of PBS. The resulting suspension was applied to a poly-L-lysine-coated coverslip and incubated for 40 min to allow the cells to settle. The coverslip was then washed with PBS. All measurements were performed in PBS solution.

### 2.5. AFM

An NTEGRA BIO atomic force microscope (NT-MDT SI, Moscow, Russia) was used to visualize the cell surface. NSG01 cantilevers (NT-MDT SI, Moscow, Russia) with a probe radius of ~10 nm and a spring constant of 5 N/m were utilized. All images were acquired with a resolution of 256 × 256 or 512 × 512 pixels and a scan size ranging from 5 × 5 µm^2^ to 100 × 100 µm^2^. The lateral scan rate was ~0.3 Hz. All surface maps were collected in air at a relative humidity of 35–50%.

The elastic (Young’s) modulus of cells was derived from force-distance curves collected with NTEGRA Prima AFM (NT-MDT SI, Moscow, Russia). SD-Sphere-CONT-L-10 probes (Nanosensors, Neuchâtel, Switzerland) with a spring constant of 0.2 N/m and a tip radius of 2000 nm were used. All measurements were performed at room temperature (24 °C) in PBS solution.

The cantilever was calibrated before each experiment. Vertical deflection sensitivity was determined against a clean glass slide in PBS solution. All force-distance curves were collected at a fixed vertical approach speed of 5 µm/s. The Hertz model was utilized to analyze the force-distance curves and calculate the Young’s modulus (E).

### 2.6. Roughness

The roughness of a cell surface is a quantitative characteristic that describes the microstructural irregularities of its topography. To evaluate cell roughness, all AFM images were processed using Image Analysis version 3.5 (NT-MDT SI, Moscow, Russia), Gwyddion version 2.65 (Czech Metrology Institute, Brno, Czech Republic), and Femtoscan version 2.3.239 (Advanced Technologies Center, Moscow, Russia) [21,22,23,24]. A cut-off parameter of 5 µm was selected to maximize the number of surface features included in the assessment of local membrane roughness. This parameter excludes large-scale topographic components unrelated to the membrane’s nanostructural features, thereby focusing on functionally relevant micro-irregularities. Such an approach enhanced the method’s sensitivity to the effects of CPB on the cell-surface nanostructure and allowed for a consistent assessment of all AFM images, including those with lower resolution. It also ensured accurate comparability of the results and eliminated the influence of differences in scanning parameters on the final roughness measurements.

### 2.7. Statistical Analysis

Statistical analysis was performed using OriginPro 2019 software (OriginLab Corporation, Northampton, MA, USA). All data were evaluated for normality using the Shapiro–Wilk test (for fewer than 50 observations) or the Kolmogorov–Smirnov test (for more than 50 observations). Data exhibiting a normal distribution are presented as mean ± standard deviation (SD), while data with a non-normal distribution are presented as median (ME) and interquartile range (Q1; Q3). The Mann–Whitney test was used to compare independent samples, and the Wilcoxon signed-rank test was used for paired samples. Results were considered statistically significant at *p* < 0.05.

An additional multivariable analysis of changes in erythrocyte parameters (Δ = post − pre) was performed using multiple linear regression. For the dependent variables ΔMorph (% discocytes), ΔRtm, and ΔE, the model included the following predictors: operation type (as Group), age (as Age), and total procedure duration (as t). Full results are provided in the Supplementary Materials (Table S1).

## 3. Results

### 3.1. Morphological Changes

**Figure 2:**
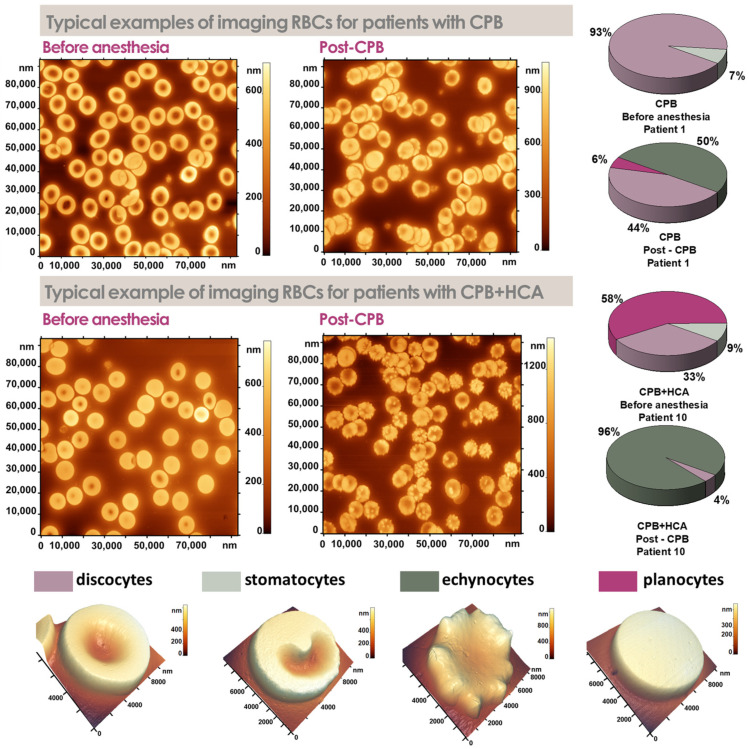
Atomic force microscopy images and the distribution of erythrocyte morphological forms in patients before anesthesia and post-CPB with cardiopulmonary bypass (CPB) or CPB combined with hypothermic circulatory arrest (CPB+HCA).

In this study, red blood cells were classified into four morphological types: discocytes, echinocytes, stomatocytes, and planocytes (Figure 2).

Morphological analysis showed that in Group 1, the proportion of discocytes decreased in most patients after surgery (Figure 2). However, for patients 3 and 4, this decrease was minimal.

Group 2 exhibited a more severe disruption of RBC morphology. In all patients in this group, discocytes were completely absent after surgery, regardless of the preoperative proportion, which ranged from 50% to 97%. Notably, in patient 9, the proportion of discocytes dropped from 74% to 0%, while echinocytes constituted 100% of the observed cell population (Figure 2).

**Figure 3:**
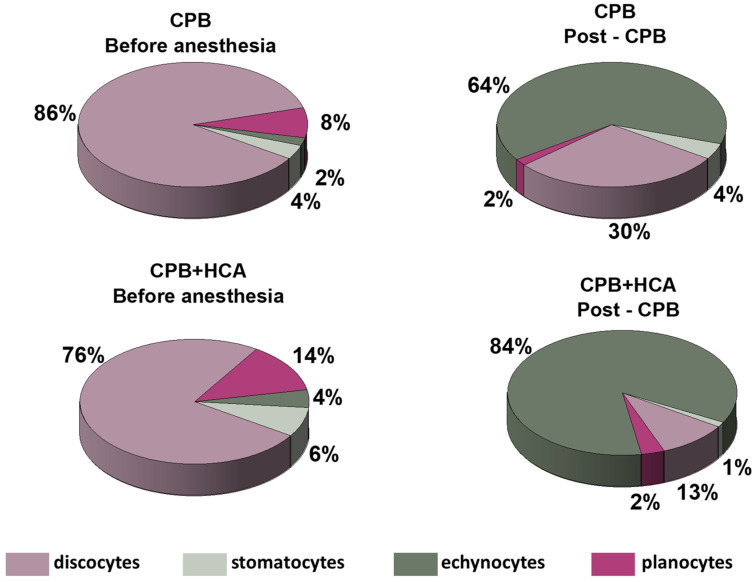
Distribution of erythrocyte morphological forms in the study groups before anesthesia and post-CPB.

A representative example of cell morphology with corresponding diagrams for all patients is presented in Figure 3. The morphological analysis indicates that procedures involving CPB+HCA induce a more pronounced transformation of red blood cells than CPB alone. This is manifested in the complete absence of discocytes and a significant increase in the proportion of morphologically altered cells.

### 3.2. Nanostructural Changes in the RBC Membrane

To evaluate nanostructural changes in the erythrocyte membrane, the average maximum height of the profile (Rtm) was analyzed. Individual analysis revealed significant inter-patient variability in the preoperative Rtm parameter baseline. Therefore, for an accurate comparison, relative changes, calculated as the ratio of the postoperative value to the preoperative baseline for each patient, were used.

Group 1 exhibited a wide range of Rtm changes, from a decrease (patient 3, 0.8-fold) to a significant increase (patient 7, 4.75-fold, *p* < 0.01). Most patients showed increased erythrocyte surface roughness, with the parameter increasing from 1.1- to 2.9-fold. Group 2 also displayed notable changes, with a maximum Rtm increase of 3.4-fold (patients 10 and 12, *p* < 0.01). A decrease in Rtm after surgery was observed in only one patient in this group (0.8-fold).

**Figure 4:**
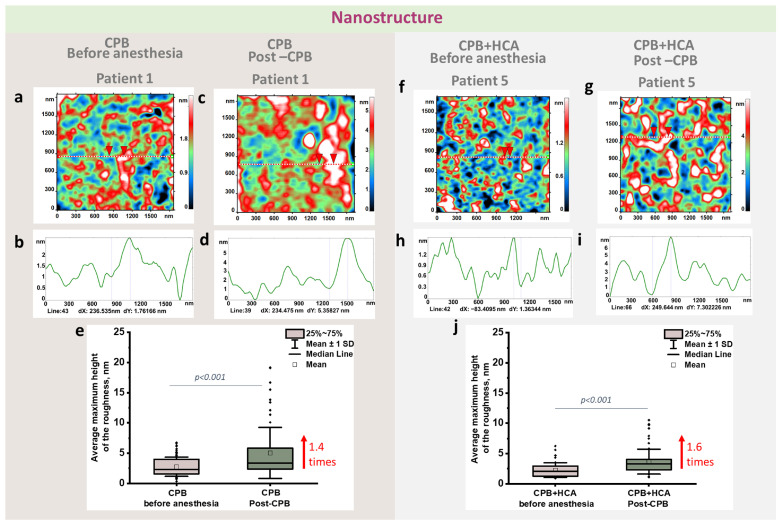
The nanostructure of erythrocyte membranes before anesthesia and post-CPB in the cardiopulmonary bypass (CPB) and CPB with hypothermic circulatory arrest (CPB+HCA) groups. (**a**,**c**) AFM images of erythrocyte membrane nanostructure in patient 1 before anesthesia and post-CPB in the CPB group; the dashed line indicates the direction used to generate the profile. (**b**,**d**) Corresponding linear height profiles. (**f**,**g**) AFM images of erythrocyte membrane nanostructure in patient 5 before anesthesia and post-CPB in the CPB+HCA group; the dashed line indicates the profile line. (**h**,**i**) Corresponding linear profiles. The color scale represents surface height (nm). Purple lines (**b**,**d**,**h**,**i**) correspond to the red triangles, which indicate the amplitude (height) of oscillations between the minimum and maximum. (**e**,**j**) Changes in the mean maximum height of erythrocyte surface roughness before anesthesia and post-CPB in CPB and CPB+HCA groups. The box indicates the 75th percentile and the whiskers indicate the mean standard deviation. The square represents the mean. Dark dots marked experimental data. Statistically significant within-group differences were assessed using the Wilcoxon signed-rank test. No statistically significant differences were found between groups.

The changes in the average maximum height of the profile of the erythrocyte membrane before and after CPB for both groups are shown in Figure 4.

When averaged, the mean Rtm increased 1.4-fold in Group 1 (*p* < 0.0001) and 1.6-fold in Group 2 (*p* < 0.0001). This indicates that more pronounced alterations of the RBC membrane nanostructure occurred in patients who underwent surgery with circulatory arrest, likely due to the combined effects of hypothermia, hemodilution, and reperfusion stress.

In addition to the quantitative analysis (Figure 4e,j) presented in Figure 4 shows representative AFM images of erythrocyte membrane nanostructure for patient 1 in the CPB group and patient 5 in CPB+HCA group, obtained before anesthesia and post-CPB, together with the corresponding cross sectional height profiles (Figure 4a–d for patient 1 and Figure 4f–i for patient 5). The linear scanning profiles demonstrate postoperative changes in membrane surface relief, which is consistent with the observed changes in the Rtm parameter.

### 3.3. Mechanical Properties

The elastic (Young’s) modulus (E) increased for all samples in Group 1 (1.1- to 1.7-fold), indicating larger stiffness of the erythrocytes after surgery. The most significant increase was observed in patient 3 (1.7-fold, *p* < 0.0001). In Group 2, the changes in E were less pronounced (1.0- to 1.3-fold). For patients 10, 11, 13, and 14, no significant increase in stiffness was observed, suggesting that cytoskeletal elasticity was preserved despite notable morphological alterations.

**Figure 5:**
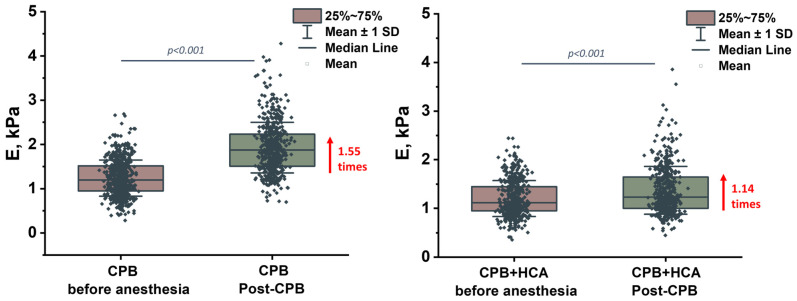
Mean Young’s modulus (E, kPa) of erythrocytes before anesthesia and post-CPB for the cardiopulmonary bypass (CPB) and CPB with hypothermic circulatory arrest (CPB+HCA) groups. The box indicates the 75th percentile and the whiskers indicate the mean standard deviation. The square represents the mean. Dark dots marked experimental data. Statistically significant within-group differences were assessed using the Wilcoxon signed-rank test. No statistically significant differences were found between groups. The within-group comparisons showed significant changes (*p* < 0.0001).

Figure 5 shows boxplots of the mean Young’s modulus values for all groups. In Group 1 (CPB), the value increased 1.55-fold (*p* < 0.0001), whereas in Group 2 (CPB+HCA), it increased only 1.14-fold (*p* < 0.0001). This indicates a relative preservation of the elastic properties of the cytoskeleton alongside more significant damage to the membrane surface under conditions of circulatory arrest.

### 3.4. Laboratory Parameters

This section presents the main laboratory parameters characterizing the degree of hemodilution and the oxygenation status of blood, measured before anesthesia and after completion of cardiopulmonary bypass. As shown in Table 1, changes in hematocrit (Hct), total hemoglobin (tHb), and oxyhemoglobin fraction (FO_2_Hb) were comparable between the groups. No statistically significant between-group differences in the extent of hemodilution were found (*p* > 0.05).

## 4. Discussion

This study examined changes in the structural and mechanical properties of erythrocytes following cardiac surgery with CPB or with CPB combined with HCA. Morphological, surface roughness, and Young’s modulus analyses of RBCs were performed, followed by a comparison between the CPB and CPB+HCA groups. This approach enables the identification of cellular injury mechanisms that might remain hidden when relying solely on whole blood parameters, thereby broadening our understanding of the pathophysiological effects of CPB and HCA. The results of the present study demonstrate a trend suggesting that the effect of cardiopulmonary bypass on erythrocytes depends on perfusion conditions, including the presence of circulatory arrest and hypothermia. The observed changes involve erythrocyte morphology, membrane surface characteristics, and the mechanical properties of the cells.

In both groups, the proportion of morphologically altered red blood cells increased after surgery, but the changes were more significant in Group 2, driven primarily by the emergence of echinocytes and stomatocytes. In Group 1 (conventional CPB), a higher proportion of discocytes remained, indicating less severe damage. Cell surface analysis showed notable postoperative changes, which were more pronounced in Group 2 and moderate in Group 1.

In Group 1, a significant increase in the Young’s modulus was observed. This finding is consistent with known effects: erythrocyte deformability decreases under mechanical stress, hypothermia, and hemodilution, resulting in increased cell rigidity and impaired microcirculation [1,3,25,26,27]. Under CPB conditions, shear stress caused by differences in blood flow velocity plays a crucial role in cell damage. Turbulent flow within the extracorporeal circuit also contributes significantly by increasing collisions between red blood cells and artificial surfaces, leading to membrane damage.

In contrast, no significant change in Young’s modulus was observed in the CPB+HCA group despite notable membrane nanostructural alterations. A probable explanation is the application of moderate hypothermia (28 °C), which increases blood viscosity and may thereby reduce mechanical shear stress. However, this hypothesis requires further investigation. Therefore, the more pronounced morphological changes observed with CPB+HCA likely result from the combined effects of hypothermia, hemodilution, and reperfusion stress, which primarily damage the membrane surface structures of erythrocytes.

Unlike studies that have evaluated the viscoelasticity of whole blood during cardiopulmonary bypass by measuring viscosity and elasticity [25,28,29], our study employed atomic-force microscopy to examine changes at the level of individual cells. The study by Girasole et al. [28] demonstrated that whole blood viscosity significantly decreased during both normothermic and hypothermic CPB, with partial recovery 24 h after surgery. Such parameters reflect the overall interaction between plasma and cellular elements, which determines the blood’s bulk properties. In contrast, our findings revealed distinct and divergent changes at the cellular level. During conventional CPB, Young’s modulus of red blood cells increased alongside moderate surface changes. In contrast, during CPB+HCA, no significant change in Young’s modulus was observed despite pronounced alterations in membrane topology. These differences suggest that the bulk rheological characteristics of blood and the nanomechanical properties of individual cells can change independently. This dissociation should be considered when interpreting clinical data and choosing optimal perfusion strategies.

**Figure 6:**
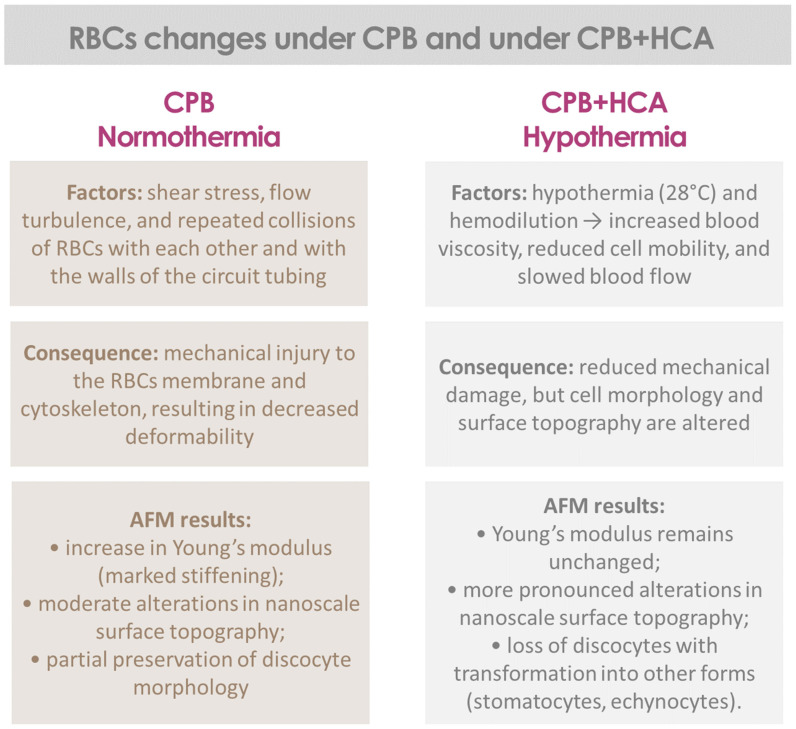
A comparative schematic of factors causing damage to red blood cells and their physiological effects during cardiopulmonary bypass (CPB) and CPB combined with hypothermic circulatory arrest (CPB+HCA).

The collected data enable us to distinguish between different mechanisms of red blood cell damage during CPB and CPB+HCA. These mechanisms and their manifestations are summarized in a schematic diagram (Figure 6).

The main limitation of the present study is its small sample size, as it is a pilot study. We are currently continuing patient enrollment in the studied groups and forming additional groups to conduct a larger study and subsequently confirm the observed patterns. In this study, in-hospital clinical outcomes and the occurrence and volume of blood transfusions were not analyzed, which limits the ability to draw direct conclusions about the association between AFM-derived erythrocyte characteristics and clinical events in cardiac surgery patients. Additional limitations include the lack of assessment of the effects of blood contact with foreign surfaces of the extracorporeal circuit, as well as ultrafiltration processes, which may potentially alter erythrocyte morphology and mechanical properties and thus affect the interpretation of the observed changes. Cardiotomy suction from the open surgical field may involve blood-air contact and could potentially affect erythrocyte status. However, within the present study, this factor could not be quantified and is therefore considered a limitation.

## 5. Conclusions

Thus, under CPB conditions without HCA, the primary changes occur in the nanomechanical properties of the cells, whereas under CPB+HCA conditions, the main transformations are observed in cell morphology and membrane topology. These differences indicate distinct damage mechanisms and highlight the importance of a comprehensive analysis of red blood cell membrane condition under various perfusion strategies.

## Figures and Tables

**Table 1:** Change in hematocrit, hemoglobin, and oxyhemoglobin fraction before anesthesia and after cardiopulmonary bypass.

	Group 1		Group 2	
	*Before anesthesia*	*Post-CPB*	*Before anesthesia*	*Post-CPB*
Hct	37.8 (35.9; 39)	27.6 (25.8; 32.1)	35.9 (35; 40.5)	26.5 (26.1; 27.3)
tHb (g/L)	122.5 (116; 127)	89 (83; 104)	117 (114; 132)	85 (84; 88)
FO_2_Hb (%)	95.9 (95.4; 97.3)	96.9 (95.6; 97.4)	97.2 (96.5; 97.3)	97 (96.8; 97.4)

## Data Availability

The datasets used and analyzed during the current study are available from the corresponding authors upon request.

## Supplementary Materials

The following supporting information can be downloaded at https://www.mdpi.com/article/10.3390/jcm15041435/s1. Table S1: Multivariable linear regression of changes in erythrocyte parameters (Δ = post − pre).

## Abbreviations

The following abbreviations are used in this manuscript:

AFM	Atomic force microscopy
E	Young’s modulus
CPB	Cardiopulmonary bypass
HCA	Hypothermic circulatory arrest
RBCs	Red blood cells
