# Supplementary material for: The Impact of Cardiopulmonary Bypass on the Structure and Mechanics of Red Blood Cells: Pilot Study
Source: J Clin Med. 2026 Feb 12;15(4):1435. doi: 10.3390/jcm15041435 (PMC12941588; doi:10.3390/jcm15041435)
Supplement: Supplementary file 1 [file jcm-15-01435-s001.zip › jcm-4082847-supplementary.pdf]

Table S1. Multivariable linear regression of changes in erythrocyte parameters (Δ = post−pre)

Dependent variable	Predictors	Value (SE)	p (parameter)	R^2 (Adj. R^2)	p (model)
ΔMorph	Group	40.026 (44.011)	0.385	0.287(0.073)	0.316
	Age	1.520 (1.852)	0.431		
	t	-0.170 (0.234)	0.484		
ΔRtm	Group	3.285 (2.254)	0.176	0.181(-0.064)	0.115
	Age	-0.069 (0.095)	0.485		
	t	-0.014 (0.012)	0.275		
ΔE	Group	-0.184 (0.405)	0.659	0.433 (0.263)	0.553
	Age	-0.024 (0.017)	0.188		
	t	-0.001 (0.002)	0.631		
